# Stated-preference research in HIV: A scoping review

**DOI:** 10.1371/journal.pone.0224566

**Published:** 2019-10-30

**Authors:** John M. Humphrey, Violet Naanyu, Katherine R. MacDonald, Kara Wools-Kaloustian, Gregory D. Zimet

**Affiliations:** 1 Department of Medicine, Indiana University, Indianapolis, Indiana, United States of America; 2 Department of Behavioral Sciences, Moi University, Eldoret, Uasin Gishu County, Kenya; 3 AMPATH Program, Eldoret, Uasin Gishu County, Kenya; 4 Department of Pediatrics, Indiana University, Indianapolis, Indiana, United States of America; University of Toronto, CANADA

## Abstract

Discrete choice experiments (DCE), conjoint analysis (CA), and best-worst scaling (BWS) are quantitative techniques for estimating consumer preferences for products or services. These methods are increasingly used in healthcare research, but their applications within the field of HIV research have not yet been described. The objective of this scoping review was to systematically map the extent and nature of published DCE, CA, and BWS studies in the field of HIV and identify priority areas where these methods can be used in the future. Online databases were searched to identify published HIV-related DCE, CA and BWS studies in any country and year as the primary outcome. After screening 1,496 citations, 57 studies were identified that were conducted in 26 countries from 2000–2017. The frequency of published studies increased over time and covered HIV themes relating to prevention (n = 25), counselling and testing (n = 10), service delivery (n = 10), and antiretroviral therapy (n = 12). Most studies were DCEs (63%) followed by CA (37%) and BWS (4%). The median [IQR] sample size was 288 [138–496] participants, and 74% of studies used primary qualitative data to develop attributes. Only 30% of studies were conducted in sub-Saharan Africa where the burden of HIV is highest. Moreover, few studies surveyed key populations including men who have sex with men, transgender people, pregnant and postpartum women, adolescents, and people who inject drugs. These populations represent priorities for future stated-preference research. This scoping review can help researchers, policy makers, program implementers, and health economists to better understand the various applications of stated-preference research methods in the field of HIV.

## Introduction

Discrete choice experiments (DCE), conjoint analysis (CA) and best-worst scaling (BWS) are quantitative methods for estimating individuals’ stated preferences for products and services [1–4]. These methods, which are based in economic/marketing theory, have been used widely in research pertaining to marketing, transportation, the environment, and other fields [5]. DCE, CA and BWS have also been applied to health economics and medical research to elicit preferences from patients, providers and policy makers, covering broad themes such as delivery of health services, resource allocation, development of outcome measures, prophylaxis and treatment products, and employment [6–10].

Stated-preference methods have also expanded into the field of HIV research, having been used to elicit preferences and attitudinal obstacles for HIV testing, service delivery, antiretroviral treatment (ART) and HIV prevention products [11–17]. Given the increasing emphasis on patient-centered approaches to HIV care delivery that are adaptive to health systems and their resource constraints, the potential for stated-preference research to address priority HIV research areas is significant [18–21]. However, it is unclear how DCE, CA and BWS methodologies have been applied in the HIV research field to date and what priority areas exist in which these methods can best be used to better understand the HIV epidemic. The objective of this scoping review was to systematically map the extent and nature of published DCE, CA, and BWS studies in the field of HIV research and identify priority areas where these methodologies can be used in the future. We chose to focus on HIV in light of the magnitude of the global HIV epidemic which has resulted in a substantial and diverse body of HIV-specific literature, our prior HIV research experience, and the current status of HIV as a chronic disease requiring a longitudinal, patient-centered care model that can be informed by stated-preference research.

### Synopsis of stated-preference methods

**Fig 1:**
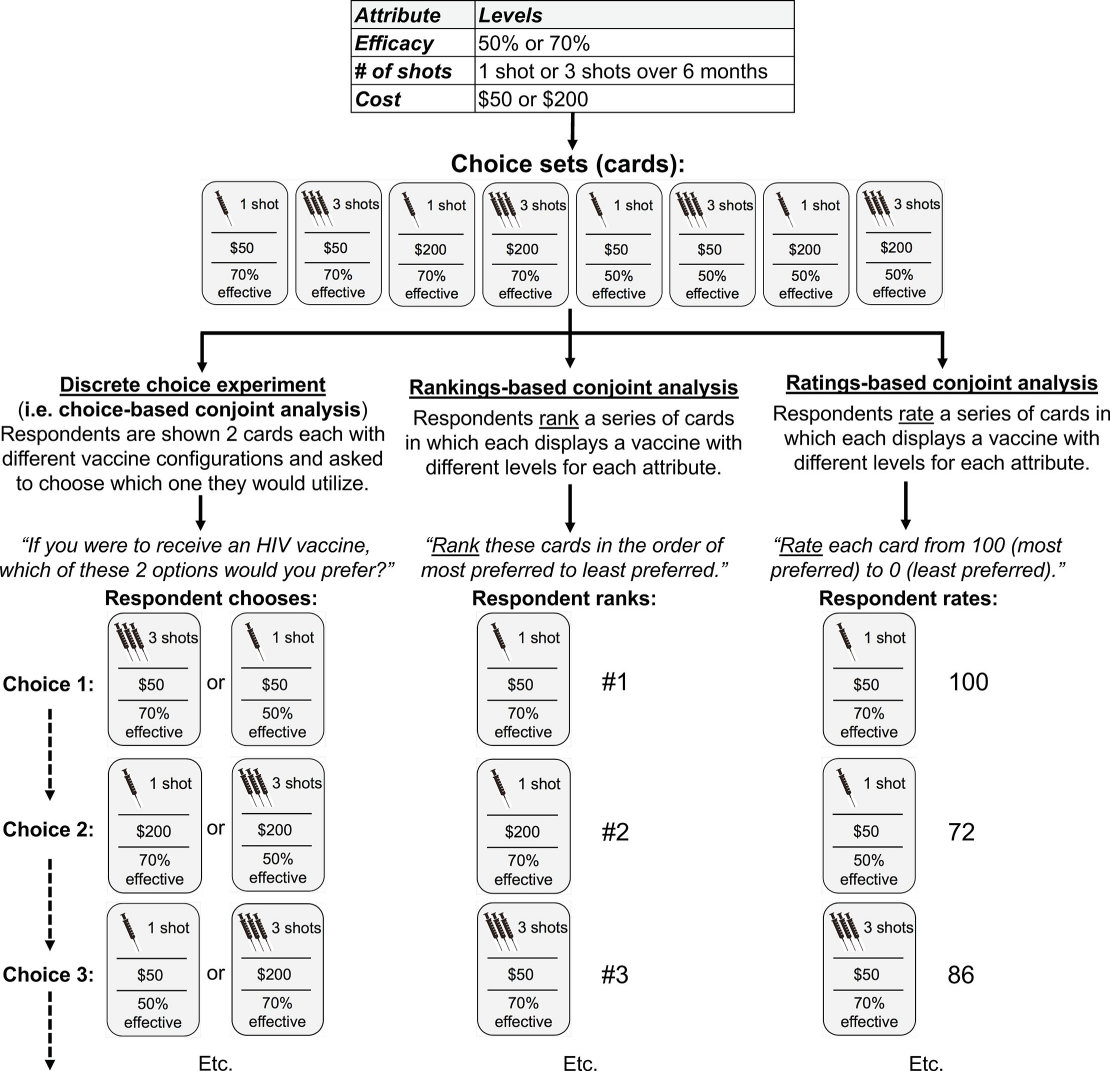
Summary of discrete choice experiment and conjoint analysis methods.

**Fig 2:**
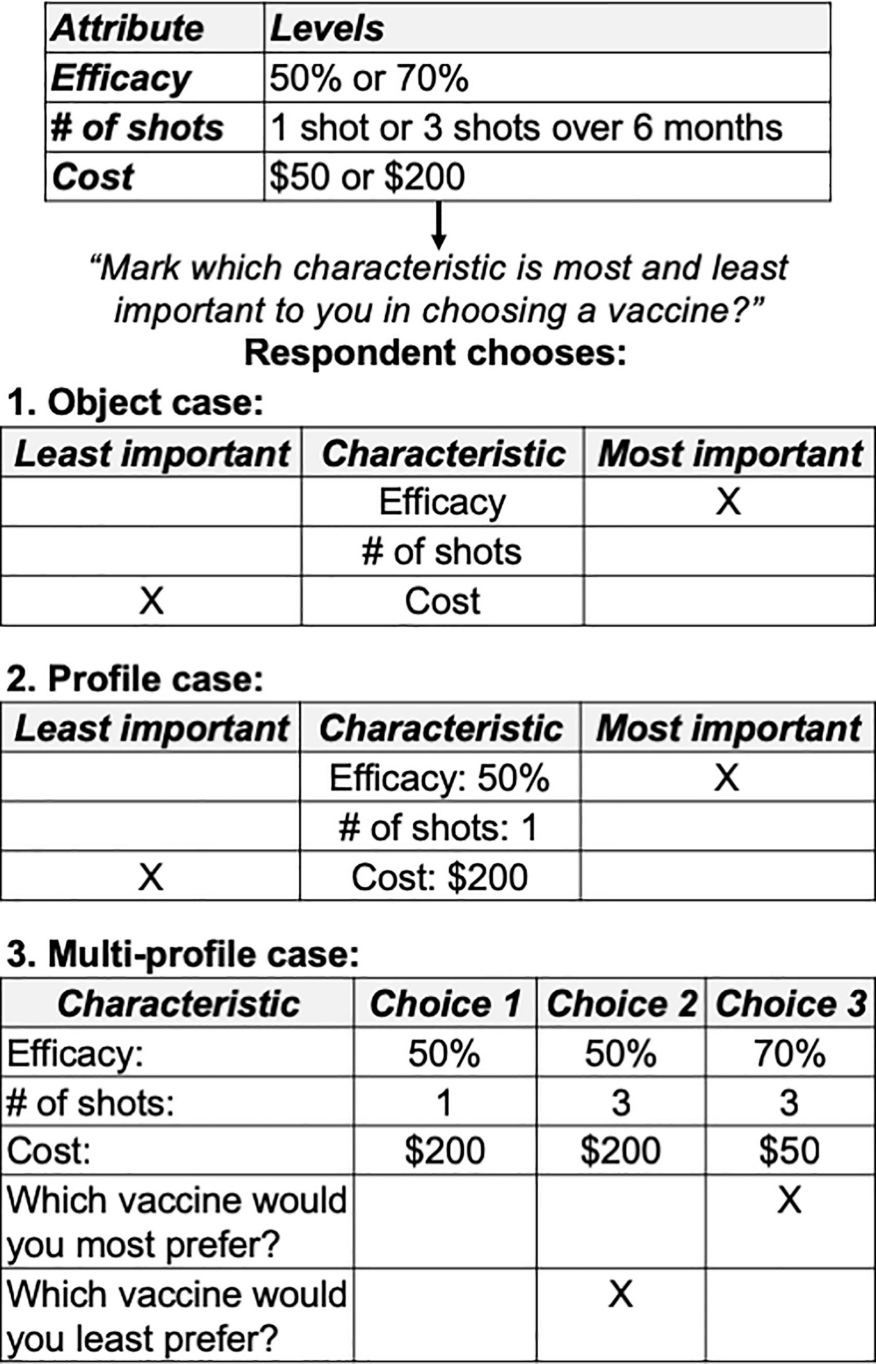
Summary of best-worst scaling methods.

There are several key steps and concepts underpinning stated-preference research methods, which are reviewed in depth elsewhere and summarized here as follows (Fig 1) [7, 20]. First, DCE, CA and BWS are subtypes of stated-preference research that utilize *conjoint measurements* (i.e. measurements taken on all parameters simultaneously) to draw comparisons between sets of defined alternatives [22]. DCE is really a distinct conjoint approach different from other CA methods although it is sometimes referred to as choice-based CA [23]. In our review, we use CA to refer to non-discrete choice conjoint analytic approaches. (Contingent valuation is another subtype of stated-preference research that is not discussed in this review.) CA methods include ranking and ratings-based subtypes. In ratings-based CA, each scenario is rated independently (in theory) of the other scenarios, while in rankings-based CA, the evaluations are not independent of each other. In DCE, respondents consider multiple scenario configurations simultaneously, and in BWS (also called MaxDiff analysis), respondents must choose the ‘best’ (i.e. most preferred) and ‘worst’ (i.e. least preferred) options among a choice task containing at least three alternatives. There are three subtypes of BWS (i.e. object, profile and multi-profile case) that differ in the complexity of the items under consideration (Fig 2) [24].

For all types of stated-preference research, the investigator must first identify and present the characteristics (known as “attributes”) that compose each hypothetical set (known as “choice sets”) that will be presented to the study participants. Each choice set contains various attributes which in turn contain various options or increments (termed “levels”). The choice sets selected by participants thus represent their preferences over other choice sets (i.e. hypothetical alternatives). In cases where the number of attributes and levels make for an unreasonably large number of unique choice sets for a participant to select from, a fractional factorial design is often employed to reduce the number of choice sets while maintaining the statistical integrity of the model [20]. Responses are then analyzed to quantify the relative importance of each attribute, how/whether the participant’s preferences are influenced by the attributes, and the trade-offs participants are willing to make between varying hypothetical alternatives [20]. As one would expect, the selection of attributes and levels is critical to study design in order to accurately reflect the preferences that would be made by a population in a real-world context [25].

## Methods

### Protocol and registration

The protocol was developed by our research team using the Preferred Reporting Items for Systematic Reviews and Meta-Analyses Extension for Scoping Reviews (PRISMA-ScR) [26]. The final protocol was registered with the Open Science Framework (https://osf.io).

### Eligibility criteria

We utilized the following eligibility criteria to broadly map the literature on stated-preference research in HIV: 1) the target study population is people living with HIV (PLWH) or persons at risk of HIV infection (as identified by the authors of the report), healthcare workers interfacing with PLWH, or policy makers addressing HIV-related issues; and 2) DCE, CA, or BWS methodology was used to elicit and analyze preferences of the study population. All published manuscripts and conference abstracts published in any year, language, or country were included. Reviews and opinion pieces were excluded.

### Outcomes

Scoping reviews are a relatively novel form of knowledge synthesis that follow a systematic approach to map the evidence on a topic to identify key concepts, theories, sources and knowledge gaps [26]. Using this approach, the primary outcomes of our scoping review were to determine the extent (i.e. number of studies), range (i.e. variety of study types), and nature (i.e. characteristics) of eligible studies.

### Data sources and search strategy

We conducted a systematic search following the Cochrane Collaboration guidelines and report our findings using the PRISMA-ScR (S1 Table) [26–28]. Our search criteria was informed by recent systematic reviews of the literature of health-related, stated-preference studies (S2 Table) [1, 2, 29–31]. We searched PubMed (indexed since 1945), Embase (indexed since 1947 and includes conference abstracts), PsycINFO (indexed since 1967 and includes conference abstracts), and the Cumulative Index to Nursing and Allied Health Literature (CINAHL; indexed since 1990), using text and MeSH terms exploded to include all subheadings. The literature search was conducted February 10, 2018 by the primary author (John Humphrey).

### Study selection

Titles and abstracts were imported into Endnote X8 (Thompson Reuters, Philadelphia, United States), duplicates were removed, and they were screened by two authors (John Humphrey and Katherine McDonald) with potential eligibility determined by consensus with a third author (Gregory Zimet) when eligibility was unclear. Full texts of potentially relevant records were retrieved and assessed for eligibility. Reference lists of all potentially eligible articles and reviews were also searched for additional titles relevant to the search, as well as to search for methodological details missing from the included report, as necessary.

### Data charting and synthesis of results

Data were extracted from eligible studies by two of the authors (John Humphrey, Katherine McDonald) and charted in using a standardized data abstraction form developed in Microsoft Excel (Redmond, WA). The form was developed by John Humphrey and Gregory Zimet for the study and designed to be consistent with other healthcare-focused, stated-preference systematic reviews [1, 32]. The following information was extracted for each study: author, country where study was conducted, study year(s) (including publication year when study year(s) were not reported), objective, stated-preference type (DCE, CA or BWS), population, use of probability sampling (i.e. any sampling method that involves some form of random selection), sample size, attribute and level determination method, number of attributes, and number of choice sets presented to participants. Studies were compiled in a table and organized by year within each of the following categories: HIV prevention, HIV counselling and testing, HIV care and service delivery, and ART. These categories were selected after completion of the systematic search and data extraction and were informed by the organizational format of the World Health Organization HIV clinical guidelines [33].

### Critical appraisal of individual studies

Eligible studies were included in the review regardless of their methodological quality or risk of bias. However, a quality assessment was conducted to gain a fuller understanding of the nature of the available evidence. Given a lack of a standard quality or bias assessment tool for stated-preference studies, a customized quality assessment tool was created utilizing domains from the Lancsar and Louviere 2008 and the Quality Assessment Tool for Observational Cohort and Cross-Sectional Studies published by the National Heart, Lung, and Blood Institute (NHLBI), and pilot tested on five random studies [3, 34]. The following domains were assessed: reported methodology concordance (e.g. that studies reporting using CA are using CA and not DCE) [23]; participation rate (< or ≥ 50%); recruitment method; whether inclusion/exclusion criteria were explicit; whether a sample size justification, power description, or variance and effect estimates were provided; design type (full or fractional factorial); whether a forced choice was used and if so, whether a justification was provided; method of profile generation and allocation to choice sets; whether respondents were randomly allocated to versions; whether coverage of attributes and levels was checked via piloting; whether understanding and complexity was checked via piloting; and, how data were collected (e.g. face-to-face, phone-, self- or computer-administered questionnaires). Checklist items that were not present in the report (either because the item in question was not performed or not reported by the authors) were scored ‘n/s’. The critical appraisal data extraction was conducted by two of the authors (John Humphrey, Katherine McDonald) for each study through a separate process following the initial data extraction. These data were then summarized in a table for all full-text articles. Although all reports were included in the critical appraisal, conference abstracts were excluded from this summary table given that the word limits of abstracts may have precluded descriptions of all of the items in our checklist.

## Results

### Search results

**Fig 3:**
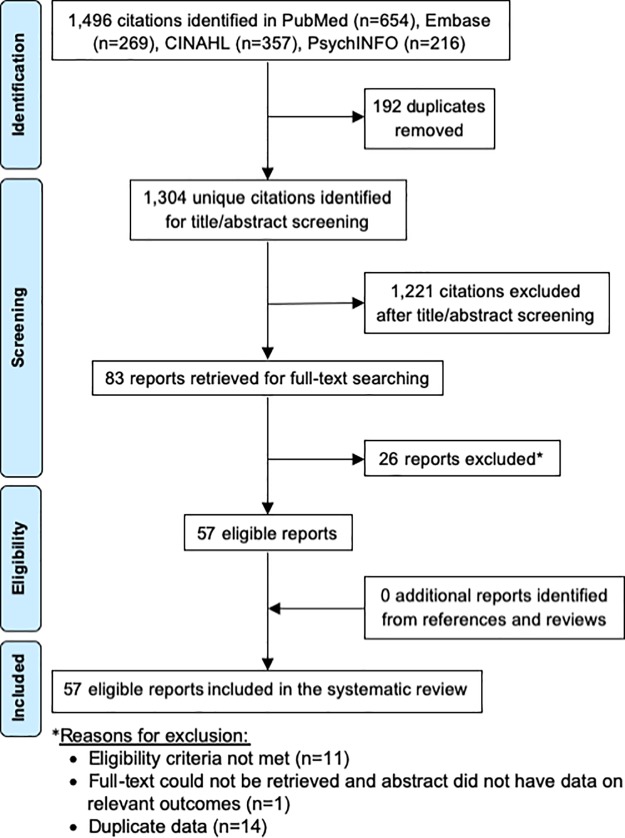
Flow diagram of article selection.

The selection process based on PRISMA guidelines is illustrated in Fig 3.[28] The search yielded 1,496 citations, 57 of which were included in the study following the screening process. Table 1 shows a summary of included studies organized by category (HIV prevention, HIV counselling and testing, HIV care and service delivery, and ART) followed by year(s) in which each study was conducted (or published, if the study year(s) were not reported), from earliest to most recent.

**Table 1:** Summary of HIV research studies using discrete choice, conjoint analysis and best-worst scaling methods (n = 57).

Author, Ref.	Country	Study Year(s)	Study Objective	Study Type	Population	Probability Sampling	Sample Size	Method of Attribute & Level Selection	Number of Attributes	Number of Choice sets
**HIV Prevention (n = 25)**										
Christofides [35]	South Africa	2003–04	To describe which aspects of health services after rape are most valued by women and the trade-offs women would make between different aspects of service delivery.	DCE	Women without HIV	n/s	319	FG, KII,	5	16
Scalone [36]	United Kingdom, USA	2004	To investigate preferences and willingness to pay for medical treatments for genital herpes.	DCE	Adults with genital herpes	Yes	154	LR	6	16
Mays [37]	USA	2004*	To examine nurses’ willingness to recommend STI vaccines to parents of adolescent patients.	CA	Nurses	No	224	LR	4	13
Terris-Prestholt [17]	South Africa	2005	To understand the relative strength of women’s preferences for new HIV prevention product characteristics.	DCE	Women without HIV	No	1,017	FG, KII	5	6
Holt [38]	USA	2006*	To assess preferences for various possible microbicide characteristics.	CA	Adults at risk of HIV	No	335	FG	6	15
Newman [39]	USA	2006–07	To assess HIV vaccine acceptability.	CA	Adults at risk of HIV	Yes	1,164	KII, FG, LR	7	9
Lee [40]	USA	2008*	To assess preferences for hypothetical HIV vaccines.	CA	Adults without HIV	No	27	AT, FG, KII, LR	7	8
Tanner [41]	USA	2008*	To assess preferences for microbicide characteristics.	CA	Adolescents and young women	No	405	AT, FG, KII	4	8
Reese [42]	USA	2008–09	To evaluate patients’ preferences for accepting a kidney from a donor with an increased risk of having a blood-borne viral infection such as HIV.	DCE	Adults kidney transplant candidates without HIV	No	175	KII, FG, LR	3	12
Cameron [43]	Thailand	2008–09	To estimate the marginal willingness-to-pay for attributes of a hypothetical HIV vaccine.	CA, BWS, DCE	Adults without HIV	No	324	FG, LR	7	8
Newman [44]	Thailand	2008–09	To assess HIV vaccine acceptability, the impact of vaccine attributes on acceptability, and risk compensation intentions.	CA	MSM and transgender women	No	255	KII, LR	7	8
Eisingerich [45]	Peru, Ukraine, Kenya, Uganda, Botswana, South Africa, India	2010–11	To explore attitudes and preferences towards attributes of PrEP programs and medications, and the future acceptability of PrEP.	DCE	Female sex workers, MSM, serodiscordant couples	No	1,824	KII	5	10
Wheelock [46]	Thailand	2011	To examine the attitudes, preferences and acceptability of PrEP.	DCE	MSM without HIV	No	260	KII	5	10
Galea [47]	Peru	2011*	To examine PrEP acceptability.	CA	Female sex workers, MSM, transgender persons	No	45	KII, LR	7	8
Kinsler [48]	Brazil, Peru	2012	To explore the acceptability hypothetical rectal microbicides.	CA	MSM	No	128	FG, KII, LR	7	8
Lee [49]	USA	2012*	To assess future HIV vaccine acceptability.	CA	Adults at risk of HIV	No	143	FG, KII	7	8
Bridges [50]	South Africa	2012*	To value design characteristics of potential community-based male circumcision services to prevent HIV transmission.	DCE	Community members	Yes	645	KII	11	6
Newman [51]	Thailand	2013	To assess preferences and acceptability of hypothetical rectal microbicides.	DCE	MSM, transgender women, sex workers	No	408	AT, FG, LR	5	8
Tang [52]	Peru	2014	To understand the acceptability of hypothetical rectal microbicides.	CA	MSM	No	1,008	LR	6	8
Quaife [53]	South Africa	2015	To explore preferences regarding HIV prevention products, quantify the importance of product attributes, and predict uptake of products.	DCE	Adults, adolescent girls, Female sex workers	Yes	609	FG, LR	6	10
Shrestha [54]	USA	2016	To investigate PrEP acceptability and preferences for PrEP delivery.	CA	People who use drugs	No	400	AT, FG, KII, LR	6	8
Dubov [55]	Ukraine	2016	To determine preferences for PrEP delivery.	DCE	MSM	No	1,184	KII, LR	5	14
Primrose [56]	USA	2016*	To better understand what sexually-active women want in a vaginal microbicide to protect against HIV transmission.	CA	Women without HIV	No	302	n/s	7	49
Alcaide [57]	Zambia	2016*	To identify the importance of factors underlying the decision to engage in intravaginal practices that may increase the risk of HIV transmission.	CA	Women with HIV	No	128	FG, KII	3	9
Rodriguez [58]	Zambia	2017*	To explore the importance of factors underlying women’s decisions to engage in intravaginal practices that may increase HIV acquisition risk.	CA	Women without HIV	No	84	FG, KII	3	9
**HIV Counselling and Testing (n = 10)**										
Phillips [59]	USA	1999–2002	To examine preferences for HIV test methods.	DCE	Adults obtaining HIV tests	No	365	FG, LR, KII, pilot survey	6	11
Llewellyn [60]	United Kingdom	2011	To assess preferences for sexually transmitted infection testing services.	DCE	University students	No	233	FG, KII, LR	6	16
Lee [61]	USA	2011	To examine MSM preferences for HIV testing scenarios that influence the willingness to test for HIV.	CA	MSM without HIV	n/s	75	LR	7	8
Ostermann [62]	Tanzania	2012–14	To compare HIV testing preferences of female bar workers and male Kilimanjaro mountain porters to community members.	DCE	Adults at risk of HIV	Yes	621	FG, KII	5	9
Ostermann [12]	Tanzania	2012–13	To evaluate factors that influence HIV-testing preferences.	DCE	Adults at risk of HIV	Yes	486	FG, KII, LR, pilot survey	5	9
Bristow [63]†	Haiti	2014	To identify factors associated with willingness to test for HIV and syphilis.	CA	Adults without HIV	n/s	298	LR	6	8
Strauss [11]	Kenya	2015	To identify preferences for HIV testing service delivery models.	DCE	Male truck drivers without HIV	Yes	305	n/s	6	8
Strauss [64]	South Africa	2016*	To examine preferences for HIV counselling and testing service package characteristics.	DCE	Adolescents	No	248	FG, LR	7	16
Zanolini [65]	Zambia	2017*	To assess the attitudes and preferences for HIV self-testing.	DCE	Adolescents and adults without HIV	Yes	1,617	KII	3	9
Indravudh [66]	Malawi, Zimbabwe	2017*	To identify preferences for HIV self-testing delivery characteristics.	DCE	Adolescents and youth without HIV	Yes	138	FG, KII, LR	6	6
**HIV care and service delivery (n = 10)**										
Albus [67]	Germany	2001	To explore preferences regarding medical and psychosocial support to increase ART adherence.	CA	PLWH	No	231	KII	9	9
Opuni [68]	South Africa	2006	To measure preferences for ART clinics characteristics.	DCE	PLWH	No	1,287	FG, KII, LR	4	20
Baltussen [69]	Ghana	2006*	To determine the relative importance of different criteria in identifying priority interventions for HIV and other diseases in Ghana.	DCE	Policy makers	No	30	KII, LR	6	12
Youngkong [70]	Thailand	2010*	To determine preferences on the relative importance of criteria for priority setting of HIV programmes in Thailand.	DCE	Policy makers, PLWH, adults without HIV	No	74	FG	3	16
Michaels-Igbokwe [71]	Malawi	2012	To examine preferences for integrated family planning and HIV services.	DCE	Adolescents	Yes	524	FG, KII, LR	6	12
Kruk [13]	Ethiopia, Mozambique	2014	To identify preferences for attributes of outpatient visits for ART in the context of lifelong care.	DCE	Women with HIV	Yes	2,090	FG, KII, LR	6	8
Miners [14]	United Kingdom	2014–15	To understand which aspects of general practitioner and HIV clinic appointments PLWH most value when seeking advice for new health problems.	DCE	PLWH	No	1,106	FG, LR	7	12
Kennedy [72]†	Canada	2015*	To evaluate pregnancy-planning choices.	DCE	PLWH and people affected by HIV	n/s	25	n/s	5	n/s
Jones [73]	USA	2016*	To explore the importance of attributes involved in reproductive decision-making.	CA	Women with HIV	n/s	49	KII	5	12
Safarnejad [74]	Vietnam	2017*	To elicit preferences and trade-offs made between different HIV programs by relevant stakeholders and decision-makers.	DCE	Policy makers	No	69	KII, LR	5	8
**Antiretroviral therapy (n = 12)**										
Stone [75]	USA	2002	To assess PLWH perceptions of the impact on adherence of various attributes of ART and to compare ART regimens based on patients’ perceptions of their likelihood to promote adherence.	CA	PLWH	n/s	299	n/s	10	21
Sherer [76]	USA	2005*	To assess patient preferences toward ART regimen attributes.	DCE	PLWH	No	387	n/s	9	5
Hauber [77]	USA	2006–07	To estimate the relative importance of short-term and long-term adverse event risks in exchange for virologic suppression.	DCE	PLWH	No	147	FG, KII, pilot survey	5	24
Beusterien [15]	Germany, USA	2007*	To examine preferences for ART attributes.	DCE	PLWH	No	288	LR	13	8
Muhlbacher [78]	Germany	2009–10	To examine patient preferences for HIV treatment including effectiveness, quality of life, and further treatment options.	DCE	PLWH	No	218	LR	6	8
Muhlbacher [79]	Germany	2010	To compare patient and physician perspectives of aspects of HIV treatment quality such as effectiveness, quality of life and further treatment options.	DCE	Physicians	No	131	LR	6	8
Lloyd [80]†	United Kingdom	2013*	To elicit patient and physician preferences for ART.	CA	PLWH and physicians	n/s	325	KII, LR	8	n/s
Bregigeon-Ronot [81]	France	2014	To elicit preferences for attributes of ART.	DCE	PLWH	No	101	KII	7	19
Orme [82]†	United Kingdom	2014	To estimate the strength of patient preferences for simplified ART regimens.	DCE	PLWH	No	278	KII	4	12
Gazzard [16]†	France, Germany, Spain, Italy, United Kingdom	2014*	To examine preferences for ART characteristics.	DCE	PLWH	n/s	1,582	KII, LR	7	n/s
Bayoumi [83]†	Canada	2015*	To elicit preferences about the relative importance of various attributes of ART.	DCE	PLWH	n/s	127	n/s	6	16
Hendriks [84]	Colombia	2016	To rank patients’ preferred characteristics of ART.	BWS	PLWH	No	283	LR	5	16

ART, antiretroviral therapy; AT, acceptability trial; CA, conjoint analysis; DCE, discrete choice experiment; KII, key informant interview; LR, literature review; MSM, men who have sex with men; n/s, not specified; PLWH, people living with HIV; PrEP, pre-exposure prophylaxis; USA, United States of America

* Indicates year of publication when the year(s) the study was conducted were not reported.

† Indicates conference abstract.

### Characteristics of studies

**Fig 4:**
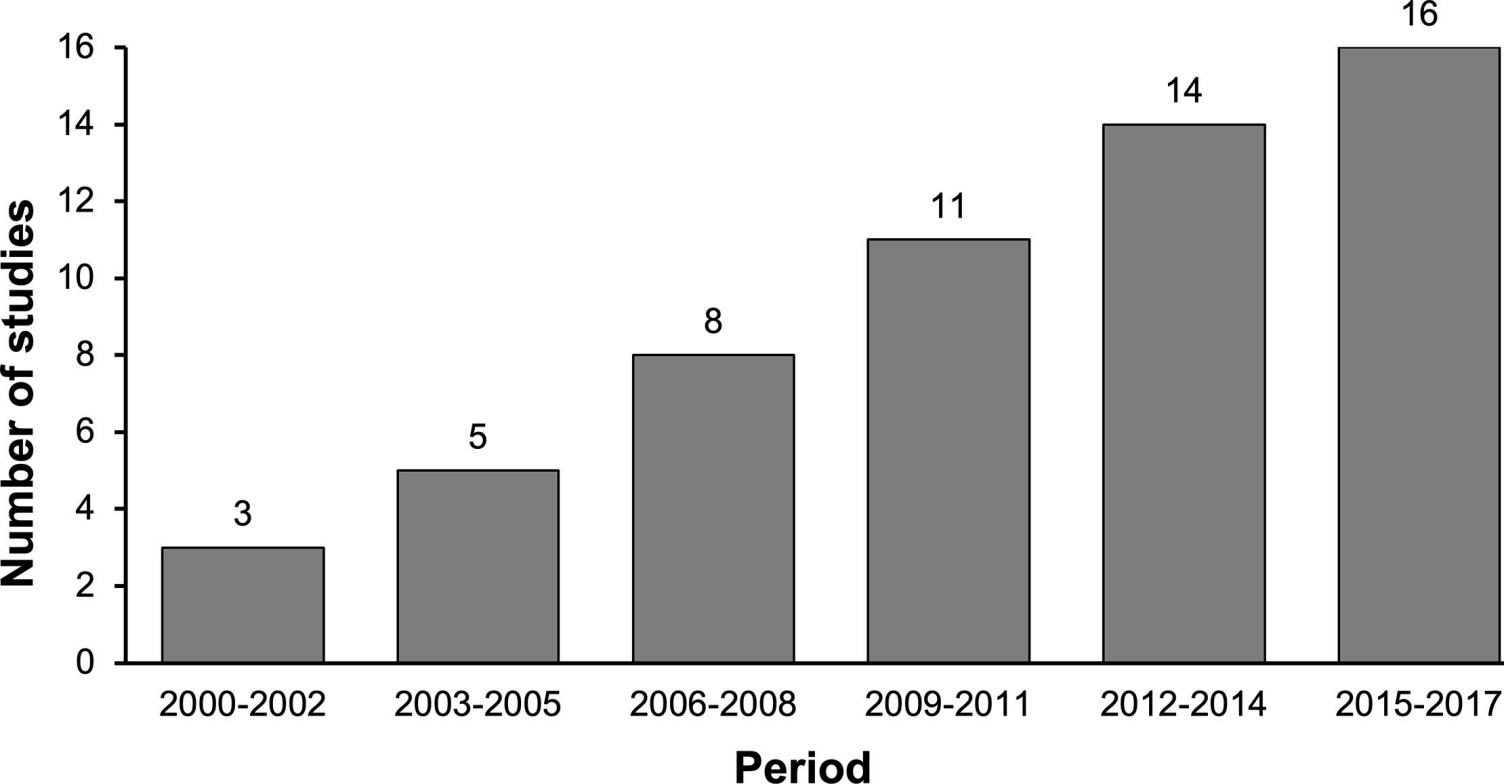
Frequency of stated-preference studies published during three-year periods from 2000 to 2017.

Studies were conducted in 27 countries from 2001–2017, with the majority conducted in the United States (30%) and South Africa (12%), while Germany, United Kingdom, and Thailand each contributed 5–6 (9–11%) studies (Table 2). The frequency of published studies reported during three-year periods from 2000 to 2017 also increased over time (Fig 4). The largest proportion of studies covered themes relating to HIV prevention (44%), while other studies covered HIV counselling and testing (17.5%), HIV care and service delivery (17.5%), and antiretroviral therapy (21%). DCE, CA and BWS methods were used in 63%, 37% and 4% of studies, respectively, and most studies did not report using any form of probability sampling. PLWH comprised 35% of respondent groups. People who were HIV-negative but considered at risk of HIV were the most common population studied overall (46%) and included adolescents and young women, members of serodiscordant couples, adults with other sexually-transmitted diseases, and male truck drivers. Participant groups from key populations included men who have sex with men (MSM), female sex workers, transgender women, and people who use drugs. The median sample size was 288 participants, and 59% of studies sampled between 100 and 500 participants. The most common methods of attribute determination were literature review and key informant interviews; 56% of studies reported using ≥ 2 methods to determine attributes. The number of attributes generated ranged from 3 to 13, with 74% of studies selecting between 5 and 7 attributes. The number of choice sets ranged from 3 to 49, with 79% administering 8–16 choice sets.

**Table 2:** Characteristics of included studies (n = 57).

Characteristic	n (%)
Study type^a^	
Discrete choice experiment	36 (63%)
Conjoint analysis	21 (37%)
Best-worst scaling	2 (4%)
Region^a^	
North America	16 (28%)
South America and Caribbean	4 (7%)
Europe	14 (25%)
Asia	7 (12%)
Africa	17 (30%)
Participants^a^	
People living with HIV	20 (35%)
People without HIV^b^	26 (46%)
Female sex workers	4 (7%)
Men who have sex with men	8 (14%)
Transgender women	3 (5%)
Healthcare workers	3 (5%)
Policy makers	3 (5%)
Probability sampling	
Yes	11 (19%)
No	37 (65%)
Not specified	9 (16%)
Sample size, median (IQR)	288 (138–496)
Sample size	
< 100	9 (16%)
100–250	15 (26%)
251–500	19 (33%)
> 500	14 (25%)
Attribute and level determination^a^	
Literature review	33 (58%)
Key informant interviews	35 (61%)
Focus groups	27 (47%)
Other^c^	7 (12%)
Not specified	6 (11%)
Attributes, mean (range)	6 (3–13)
Choice sets, mean (range), n = 52	12 (5–49)

^a^ Percentages exceed 100% because some studies were conducted in > 1 field.

^b^ Includes adolescents, adults, members of serodiscordant couples, and male truck drivers.

^c^ Includes acceptability trial (n = 4) and pilot survey (n = 3).

### HIV prevention

Studies covered a wide diversity of general and key populations, including healthcare workers [37], women [17, 38, 56–58], families [45, 50], adolescents [53, 85], MSM [45, 46, 48, 51, 52, 55], transgender people [47, 51–53], female sex workers [45, 47, 51], people who use drugs [54], and immigrants [40]. Eight studies addressed HIV prevention technologies and services, including female preferences for vaginal microbicides [17, 38, 41, 53, 56] and MSM, transgender people, and sex worker preferences for rectal microbicides [48, 51, 52]. The main drivers of prevention product uptake in these studies included HIV prevention product effectiveness [17, 38, 48, 52, 53], pregnancy prevention (vaginal products only) [17, 38, 41], cost [17, 38], absence of side effects [41], and multipurpose protection against sexually transmitted infections and pregnancy [53, 56]. Four studies addressed attitudes and preferences for HIV pre-exposure prophylaxis and reported diverse findings: HIV testing was the most important attribute among MSM in Thailand [46], while cost had the greatest impact on acceptability among key populations in Peru [47], MSM in Ukraine [55], and people who use drugs in the US [54]. Hypothetical vaccine acceptability was addressed in six studies [37, 40, 43, 44, 49, 85], finding that efficacy was a major driving influence on acceptability among adults in the US [85] and general populations, MSM, and transgender people in Thailand [43, 44, 49]. Other studies included patient preferences for genital herpes treatment and the risk of HIV [36], accepting a kidney from donors at risk of HIV [42], male circumcision [50], HIV prevention services for women who have been raped [35], and intravaginal practices among women in Zambia that may increase their risk of acquiring HIV [57, 58].

### HIV counselling and testing

Studies in this category evaluated patient preferences for HIV testing attributes, identifying strong preferences for the location of testing [11, 12, 61–64, 66, 86], test method [11, 62, 63, 65, 86], timing and results [11, 61, 63, 86], accuracy [64], confidentiality [64, 66, 86], cost [11, 61, 63, 65, 86], and comprehensiveness of testing such as the availability of counselling, ART, and tests for other sexually transmitted infections [12, 60, 62, 65, 86]. Studies covered a variety of groups and regions including MSM and students in the US [60, 61], bar workers in Tanzania [62], students in South Africa [64], truck drivers in Kenya [11], and adolescents and adults in Zambia [65].

### HIV care and service delivery

The majority of studies in this category were conducted in low and middle-income countries including South Africa, Ghana, Thailand, Malawi, Ethiopia, Mozambique, and Vietnam. Four studies assessed reproductive health preferences, including the attributes that influence facility choice among HIV-infected women of childbearing age in Ethiopia and Mozambique [13], and fertility planning for women, youth, and others affected by HIV [71–73]. Other studies assessed how to configure health services for PLWH more generally. In one study from South Africa, cost, staff attitude, wait time, and clinic branding constituted major barriers to ART uptake and adherence in resource-poor settings [68]. In the United Kingdom, preferences for shorter appointment waiting times, longer opening hours, and the type of HIV care provider (general practitioner vs HIV clinic) were prioritized [14], while flexible medical and psychosocial support were priority features according to policy makers and PLWH in Thailand [70]. Three studies specifically assessed preferences of policy makers regarding HIV program design, identifying preference for prevention interventions [74] among high-risk groups [70] and compared to other non-HIV related public health interventions [69].

### Antiretroviral therapy

Eleven studies addressed patient and physician preferences for various attributes of ART, all of which were conducted in high-resource settings in North America and western Europe. Many of these studies addressed ART attributes that would optimize adherence and quality of life for patients, finding that avoiding major side effects [15, 16, 75, 77, 81–83], long-term safety [16, 80, 82], treatment effectiveness [15, 16, 81], limited drug-drug interactions [81], and regimen convenience (e.g. tablet count and size, co-formulated tablets, dosing frequency) [15, 75, 76] were among the most important drivers of treatment choice for patients. Overall, the choice of ART was highly affected by patient preferences, with the majority of attributes studied being important to patients to varying degrees.

### Critical appraisal of individual studies

The author-reported methodology was not concordant in 20% of studies, which in all cases were presented as CA but instead described a DCE (Table 3 and S3 Table). The eligibility criteria were explicitly reported in 69% of studies, while 10% of studies provided any sample size justification, power description, or variance and effect estimate. The majority (88%) of studies used a fractional factorial design and most (65%) did not specify whether a forced choice was used. The method of profile generation and allocation to choice sets was specified in 41% of studies, with Sawtooth (Provo, UT), SPSS (Armonk, NY), Ngene (Sydney, Australia) and SAS (Cary, NC) software used in 73% of these studies. Face-to-face interviews were used to collect data in 73% of studies, followed by web surveys (16%) and self-administered questionnaires (12%). Understanding and complexity of the questionnaire was checked (e.g. through piloting) in 47% of studies, and 59% of studies did not report that participants were randomly allocated to versions of the questionnaire.

**Table 3:** Critical appraisal of full-text articles (n = 51).

Characteristic	n (%)
Methodology reported was concordant	
Yes	41 (80%)
No	10 (20%)
Eligibility criteria was explicit	
Yes	35 (69%)
No	16 (31%)
Sample size justification, power description or variance and effect estimates provided	
Yes	5 (10%)
No	46 (90%)
Factorial design	
Full factorial	6 (12%)
Fractional factorial	45 (88%)
Forced choice used	
Yes	5 (10%)
No	13 (25%)
Not specified	33 (65%)
Method of profile generation and allocation to choice sets specified	
Yes	21 (41%)
No	30 (59%)
Method of data collection^a^	
Face-to-face interviews	37 (73%)
Web surveys	8 (16%)
Self-administered questionnaires	6 (12%)
Phone interviews	1 (2%)
Participation rate ≥ 50%	
Yes	22 (43%)
No	1 (2%)
Not specified	28 (55%)
Understanding and complexity checked	
Yes	24 (47%)
No or not specified	27 (53%)
Participants randomly allocated to versions	
Yes	21 (41%)
No or not specified	30 (59%)

^a^ Percentages exceed 100% because some studies used >1 data collection method.

## Discussion

In our scoping review of DCE, CA and BWS research pertaining to HIV, 57 studies were identified covering a variety of themes relating to HIV prevention, care and treatment across diverse settings and populations. The majority of studies were DCEs (63%) followed by CA (37%). BWS was conducted in only two studies possibly reflecting the more recent introduction of BWS in health research [31]. Our review supports the increasing applications of stated-preference methods in the field of HIV research as well as the diverse uses of stated-preference research to advance knowledge about the global HIV epidemic.

The studies in our review offer key lessons for HIV policy and service delivery. First, most studies addressed HIV prevention products, including pre-exposure prophylaxis, microbicides, and vaccines. Accelerating HIV prevention is a major target in the global HIV response, and understanding client preferences and attitudes for prevention products, which may differ across population groups, is critical to maximizing the uptake and impact of these products [53, 87]. Clinical trials of pre-exposure prophylaxis and female prevention products (e.g. microbicide gels, vaginal rings) have identified adherence as a major factor influencing these products’ effectiveness, and adherence is influenced by consumer preferences and attitudes [88]. Adherence is also a key determinant of ART effectiveness, and a number of studies examined patient attitudes and preferences towards ART. Multiple studies addressed the HIV care cascade, including HIV testing and service delivery. Reaching the UNAIDS 90-90-90 targets globally, in which 90% of people with HIV know their status, 90% of those who know their status are on ART, and 90% of those on ART achieve viral suppression, requires a thorough understanding of the barriers to achieving each of these targets in general and key populations. The stated-preference studies in our review contribute to this goal, providing insights about HIV testing preferences, key attributes of ART from the perspectives of PLWH, and preferences for HIV programs among PLWH and policy makers.

Our review sheds light on several priorities for future stated-preference research. First, only 17 of 57 (30%) studies were conducted in sub-Saharan Africa where the burden of HIV is highest, and none of these studies addressed patient preferences for ART. There is significant opportunity to conduct stated-preference studies in sub-Saharan Africa given that they are generally low risk, low cost and simple to implement. Second, as HIV care programs expand to serve increasing numbers of patients, there is a need to identify patient and provider preferences for patient-centered models of care that will improve efficiency, retention on ART, and viral suppression. Examples includes models of differentiated care for stable patients and patients requiring additional support, decentralized models of ART delivery, models of integrated HIV and maternal and child health care for pregnant and postpartum women and their HIV-exposed infants, and adolescent-friendly care delivery. Third, as novel pharmacologic agents and formulations become available for HIV treatment, prevention, and possibly even sustained HIV remission, understanding patient attitudes and preferences for these products will be needed to enable their delivery, optimize their uptake, and promote patient adherence. Fourth, key populations are a priority in the HIV response and traditional public health interventions may not be suitable to address these populations’ unique needs and experiences. This includes the need to understand HIV care preferences for older children and adolescents who are cognitively capable of participating in stated-preference research but have seldom been included in such research to date.

Finally, the critical appraisal also highlighted several quality areas that should be addressed in future stated-preference research. First, 20% of studies were misclassified as CA rather than DCE. Distinguishing between these two paradigms is important given their different approaches [23]. Second, methodological details (e.g. eligibility criteria, use of a forced choice, methods of profile generation and allocation to choice sets) were not specified in a high proportion of studies, and little more than half of studies reported checking participants’ understanding and complexity of the choice tasks through piloting. Guidelines for conducting and reporting health-related CA, DCE and BWS studies have been published to help guide investigators in these areas [3, 10, 89]. Third, 74% of studies in our review reported using primary qualitative data collection (i.e. key informant interviews and/or focus groups) to develop attributes. Attribute selection is a critical step in stated-preference research, as the integrity of the choice sets depends entirely on the attributes used. Literature review alone may not yield an accurate representation of the experiences of the target population [90]. The generalizability of a study’s findings must be considered in light of the methodology used to select attributes, as well as the method of choice set presentation (e.g. tablets, cards, drawings), sampling methodology and sample size [12]. Fourth, sample sizes varied widely (25 to 2,090 participants) and were not accompanied by a sample size justification or power description in 90% of studies. Power and minimum sample size can be difficult to calculate in stated-preference studies without precisely knowing the attributes, levels and initial estimates of the parameter values [89, 91]. Nevertheless, minimum sample size can be estimated and doing so is important so that non-significant findings can be assessed in context of whether the study had sufficient power to detect a certain outcome in the first place [91]. Finally, over half of studies in our review did not report using probability sampling, which is important to acquire preference estimates that are representative of the population. However, probability sampling may not be feasible or desirable for studying key populations such as female sex workers, MSM or people who inject drugs.

Our review has strengths and limitations. Our systematic approach enabled us to assess the extent and nature of stated-preference studies in the HIV field as well as areas for future research. We did not search grey or non-English literature which may have provided additional articles. However, we did not find additional articles through our review of the references of included articles which supports the comprehensiveness of our search. We also did not summarize measures of preference heterogeneity or other more nuanced and key outcomes (e.g. probability of uptake, willingness-to-pay, utility scores), as the objective of this scoping review was to examine the extent and nature of studies rather than synthesize their notably heterogeneous findings particularly for readers who are less familiar with stated-preference research. Finally, this scoping review was a large undertaking and our results are only current up to February 2018.

## Conclusion

Stated-preference research is emerging in the HIV field as evidenced by the increasing frequency of published studies over time. These studies cover diverse areas relating to HIV prevention, HIV counselling and testing, HIV care and service delivery, and ART. However, few studies were conducted in Sub-Saharan Africa or included key populations, which represent priorities for future research. These reviews can help researchers, policy makers, program implementers, and health economists to better understand the various applications of stated-preference research methods in the field of HIV.

## Supporting information

S1 Table Preferred reporting items for systematic reviews and meta-analyses extension for Scoping Reviews (PRISMA-ScR) checklist. (DOCX)

S2 Table Electronic search strategy used for the systematic search. (DOCX)

S3 Table Summary of critical appraisal of individual studies. (XLSX)
