# Supplementary material for: Stated-preference research in HIV: A scoping review
Source: PLoS One. 2019 Oct 30;14(10):e0224566. doi: 10.1371/journal.pone.0224566 (PMC6821403; doi:10.1371/journal.pone.0224566)
Supplement: S2 Table — (DOCX) [file pone.0224566.s002.docx]

**S2 Table.** Electronic search strategy used for the systematic search.

| **Theme** | **CINAHL, PsycINFO, and Pubmed, Search Criteria** |
| --- | --- |
| **HIV** | “HIV”[MeSH] OR “HIV” OR “Acquired Immunodeficiency Syndrome"[MeSH] OR “AIDS” |
| **Boolean Operator** | AND |
| **Discrete choice, conjoint analysis, and best-worst scaling** | “discrete choice” OR “DCE” OR “choice experiment” OR “choice modelling” OR “choice modeling” OR “stated choice” OR “conjoint” OR “part-worth utilities” OR “functional measurement” OR “paired comparison” OR “stated preference*” OR “stated-preference” OR “patient weighting” OR “patient rating” OR “patient ranking” OR “patient perspective” OR “patient priorities” OR “patient preference”[MeSH] OR “preference-based approach” OR “preference-based method” OR “trade-off* ”OR “direct assessment” OR “direct preference” OR "best worst" OR “worst best” OR “best-worst” OR “worst-best” OR “maximum difference” OR “maxdiff” |
| **Theme** | **Embase Search Criteria** |
| **HIV** | ‘human immunodeficiency virus’/exp OR HIV.mp OR ‘acquired immune deficiency syndrome’/exp OR AIDS.mp |
| **Boolean Operator** | AND |
| **Discrete choice, conjoint analysis, and best-worst scaling** | 'discrete choice' OR 'dce' OR 'choice experiment' OR 'choice modelling' OR 'choice modeling' OR 'stated choice' OR 'conjoint' OR 'part-worth utilities' OR 'functional measurement' OR 'paired comparison' OR 'stated preference*' OR 'stated-preference' OR 'patient weighting' OR 'patient rating' OR 'patient ranking' OR 'patient perspective' OR 'patient priorities' OR 'patient preference'/exp OR 'preference-based approach' OR 'preference-based method' OR 'trade-off*' OR 'direct assessment' OR 'direct preference' OR 'best worst' OR 'worst best' OR 'best-worst' OR 'worse-best' OR 'maximum difference' OR 'maxdiff' |
